# Transcriptome analysis provides new insights into the response of canine intestinal epithelial cells treated by sulforaphane: a natural product of cruciferous origin

**DOI:** 10.3389/fvets.2024.1460500

**Published:** 2024-10-02

**Authors:** Kaiqi Li, Jin Yan, Shiqi Wang, Chuyang Zhu, Qi Zhu, Sichen Lu, Ping Hu, Tadelle Dessie, In Ho Kim, Abdelkareem A. Ahmed, Hao-Yu Liu, Wael Ennab, Demin Cai

**Affiliations:** ^1^Laboratory of Animal Physiology and Molecular Nutrition, Jiangsu Key Laboratory of Animal Genetic Breeding and Molecular Design, College of Animal Science and Technology, Yangzhou University, Yangzhou, China; ^2^International Livestock Research Institute, Addis Ababa, Ethiopia; ^3^Department of Animal Resource and Science, Dankook University, Cheonan, Republic of Korea; ^4^Department of Veterinary Biomedical Sciences, Botswana University of Agriculture and Agriculture and Natural Resources, Gaborone, Botswana; ^5^International Joint Research Laboratory in Universities of Jiangsu Province of China for Domestic Animal Germplasm Resources and Genetic Improvement, Yangzhou, Jiangsu, China

**Keywords:** sulforaphane, transcriptome analysis, canine, intestine, inflammation response

## Abstract

This study presents a comprehensive transcriptome analysis of canine intestinal epithelial cells following treatment with sulforaphane (SFN), a naturally occurring compound found in cruciferous vegetables with established anti-inflammatory and antioxidant properties. Through high-throughput sequencing, we identified 29,993 genes, among which 1,612 were differentially expressed, with 792 up-regulated and 820 down-regulated in response to SFN treatment. Our analysis revealed significant enrichment of genes in pathways associated with the inflammatory response, lipid metabolism, oxidative stress response, and T-cell mediated immunity, suggesting SFN’s potential in modulating these biological processes. Notably, the PPARγ gene, which plays a crucial role in the body’s oxidative stress and inflammatory response, was highly up-regulated, indicating its possible centrality in SFN’s effects. Gene–gene interaction analysis further supported SFN’s role in alleviating inflammation through PPARγ, with key genes in oxidative stress and inflammatory response pathways showing significant correlations with PPARγ. Overall, our findings provide molecular evidence for SFN’s protective effects on canine intestinal health, potentially through the modulation of inflammatory and oxidative stress pathways, with PPARγ emerging as a critical mediator.

## 1 Introduction

Vegetables and fruits are considered rich sources of antioxidants that can scavenge oxygen-free radicals to protect cells from damage caused by oxidative stress (1). Sulforaphane (SFN) is an isothiocyanate compound derived from the cleavage of thioglucoside, and it is mainly extracted from cruciferous vegetables like broccoli and cauliflower (2). The black mustard enzyme present in the plant tissue or intestinal flora catalyzes the breakdown of sulforaphane into radish thiols when the vegetables are chewed or chopped (3). Previous studies have highlighted sulforaphane’s multiple biological activities (4), particularly its impact on intestinal health. Sulforaphane has shown anti-inflammatory properties, with its ability to modulate inflammatory responses and reduce the intestinal levels of pro-inflammatory factors such as TNFα (5). It can also inhibit inflammatory pathways by activating the anti-oxidative stress defense system, promote the regeneration and repair of intestinal cells, and maintain the homeostasis of intestinal flora (6). Furthermore, as a potent antioxidant, sulforaphane can mitigate damage to intestinal cells by promoting the expression or activity of cytoprotective proteins like Nrf2 (7). In addition, sulforaphane is thought to prevent intestinal diseases and cancer (8). Despite its wide range of benefits, the potential adverse effects of sulforaphane require further investigation. However, it is very unlikely for a normal diet that radish sulfur would be toxic (9).

As the significance of canine health continues to grow, there is a heightened need to focus on the health of dogs’ gut (10). The small intestine is the main digestive organ, where nutrients are digested, and it also plays a pivotal role in the immunoregulation of the gut (11). The small intestinal epithelial cells, which constitute the main cell type of the small intestinal mucosa, are responsible for nutrient absorption and the secretion of digestive enzymes. These cells are highly sensitive to external stimuli, such as stress, pathogenic microbial infections, and nutritional deficiencies, which can impair their function and integrity, leading to compromised intestinal structure and function (12). Therefore, protecting the epithelial cells of the small intestine in canine is essential for enhancing intestinal barrier function, preventing canine intestinal diseases, and is important for the study of intestinal health.

However, as a natural product, the effect of SFN on canine intestinal epithelial cells has not been investigated (30). Transcriptome sequencing has demonstrated the feasibility of mapping differentially expressed genes to known pathways, thereby illuminating the underlying logic of experimental outcomes (13). Consequently, to enhance our comprehension of SFN’s effects on canine intestinal epithelial cells and to delineate its functional attributes, we procured SFN in a prior study and employed transcriptomic analysis to evaluate the transcriptional modulation exerted by SFN on these cells.

## 2 Materials and methods

### 2.1 Cell culture and treatment

Canine small intestinal epithelial cells were cultured in high glucose DMEM (cytiva, China) medium containing 5% fetal bovine serum (Hyclone, USA), 10 ng/mL EGF (Tongli Haiyuan, China), 5 μg/mL Insulin (Vicente Biotech, China), 20 mM HEPES (Beyotime, China), and 1% penicillin–streptomycin (Solarbio, Beijing, China), at the incubator concentration of 37°C, 5% CO_2_. Cells were exposed to two treatment modalities: (1) the vehicle control group, and (2) the sulforaphane (SFN) treatment group, where cells were incubated with 4 μM SFN (purity ≥98%, sourced from Bidde, China) for a period of 48 h.

### 2.2 RNA sequencing

RNA was extracted from cells of Vehicle group and SFN-treated group using 1 mL trizol (Invitrogen, Waltham, MA, USA), and the quality was evaluated by Agilent Bioanalyzer 2100 system (Agilent Technologies, CA, USA). High-throughput sequencing was performed at Kidio Biotech Ltd. Sequencing was completed using the Illumina sequencing platform. Raw data were aligned to the pig gene expression reference Sscrofa11 (gff3 dataset v11.1.98 and genomic fasta dataset v11.1.98, downloaded from Ensembl). Raw data were processed using Trim Galore v0.6.5 (Babraham Bioinformatics - Trim Galore!), STAR v2.7.10b (GitHub - alexdobin/STAR: RNA-seq aligner) and rsem v1.3.3.^1^ Finally, FPKM (Fragments Per Kilobase of exon model per Million mapped fragments) was chosen for subsequent data analysis.

### 2.3 Gene enrichment analysis

The gene set enrichment analysis (GSEA v4.1.0) software was used to identify the enriched pathway profiles. In addition, statistically enriched biological processes or pathways in differentially expressed genes (DEGs) of the GO and KEGG pathways were ranked and categorized through the Metascape database^2^ and DAVID.^3^ GSEA enrichment analysis plots, KEGG enrichment bubble plots, Cnetplot volcano plots, and GO-pathway enrichment result circle plots were plotted through the online platform used for data analysis and visualization.^4^ Meanwhile, correlation analysis of differential genes was performed by STRING online web platform for functional protein interactions.^5^

## 3 Results

### 3.1 Gene expression and differential gene analysis

A total of 29,993 genes were identified in all samples, including 14,388 (47.97%) annotated genes and 15,605 (52.03%) unannotated novel genes. According to GO analysis, genes were mainly enriched in physiological features (1,216 genes), cellular structures (683 genes), molecular functions (420 genes), protein post-translational modification processes (333 genes), and biological processes (272 genes). The genes were categorized into 42 KEGG pathways, which mainly play a role in signal transduction, immune system, and endocrine system.

In the SFN-treated group relative to the Vehicle group, differential analysis using the ratio of FPKM was performed to screen for differential genes between the SFN-treated group and Vehicle groups. The differential expression thresholds of the genes were set at FC > 1.4 for up-regulated genes and FC < 0.8 for down-regulated genes. A total of 1,612 DEGs were identified, including 792 up-regulated DEGs and 820 down-regulated DEGs. The top 10 up-regulated genes were HCN3, CDC42BPG, PRRG2, STAP2, IRX3, LGR6, CKM, ARHGDIB, SLFNL1 and LOC100688918. The top 10 down-regulated genes were LOC100684967, LOC102152884, CALCRL, PON1, LAMC3, ICAM4, DCDC1, LOC477724, LRRD1 and MEF2B.

### 3.2 Analysis of pathway enrichment

**Figure 1:**
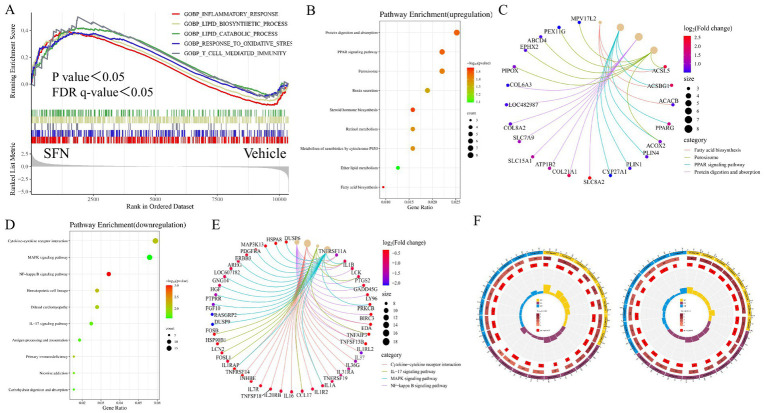
GSEA, KEGG and GO enrichment analysis of DEGs. Showing GSEA enrichment plot, enrichment bubble plot, Cnetplot and GO enrichment circle plot. **(A)** GSEA analysis showing pathways associated with differential gene expression in canine intestinal epithelial cells in the SFN group compared to controls. **(B)** KEGG enrichment analysis showing upregulated DEGs major enriched pathways, *X*-axis represents the enrichment rate and *Y*-axis represents 9 KEGG pathways. Count: bubble size indicates the number of genes annotated to KEGG pathways. *p* value: color indicates the *p* value of enrichment. **(C)** Cnetplot showing 4 pathway-related crossover genes. **(D)** KEGG enrichment analysis showing downregulated DEGs major enriched pathways. **(E)** Cnetplot showing 4 pathway-related crossover genes. **(F)** GO enrichment analysis of up- and down-regulated pathways predominantly enriched for DEGs.

To identify key transcriptional pathways regulated by SFN, transcriptome analysis was performed using canine intestinal epithelial cells from SFN-treated group (4 μM) treated and Vehicle groups. We calculated the fold change of FPKM, excluded values that were zero or nonsensical, and then enriched the remaining dataset for enrichment analysis using GSEA version 4.1.0. The genes in the Vehicle/SFN group were highly enriched in the Inflammatory response pathway, Lipid biosynthesis process pathway, Lipid catabolic process pathway, Response to oxidative stress pathway, and T-cell mediated immunity pathway (Figure 1A). Among them, the “Inflammatory response pathway” had the lowest *p* value and the highest enrichment factor. The data suggest that long-term intake of SFN can help reduce the inflammatory response of the body (14).

The up-regulated differentially expressed genes in the SFN-treated group compared to the Vehicle group were enriched in KEGG, and the enrichment was mainly concentrated in the Protein digestion and absorption pathway, PPAR signaling pathway, Peroxisome pathway, Renin secretion pathway, Steroid hormone biosynthesis pathway, Retinol metabolism pathway, Metabolism of xenobiotics by cytochrome P450 pathway, ether lipid metabolism pathway and Fatty acid biosynthesis pathway (Figures 1B,C). Cnetplot illustrated the specific genes associated with these pathways (Figure 1C). Down-regulated differentially expressed genes are centrally enriched in KEGG, including Cytokine-cytokine receptor interaction pathway, MAPK signaling pathway, NF-κB signaling pathway, Hematopoietic cell lineage pathway, Dilated cardiomyopathy pathway, IL-17 signaling pathway, Antigen processing and presentation pathway, Primary immunodeficiency pathway, Nicotine addiction and Carbohydrate digestion and absorption pathway (Figure 1D). Cnetplot shows specific genes associated with these pathways (Figure 1E).

Among the genes of these pathways PPARγ gene is highly expressed (Log2 (fold change) > 1.5) PPARγ is a nuclear receptor that plays a key role in a variety of biological processes, such as oxidative stress response and inflammatory response (15). Studies have shown that nuclear receptor PPARγ is associated with oxidative stress and directly regulates oxidative stress through the regulation of related genes involved in oxidative stress such as UCP2, GPX3, HO-1, MnSOD, and CD36, and at the same time exerts anti-inflammatory and anti-oxidative stress effects through the NF-κB signaling pathway (16).

A GO enrichment analysis was conducted. The analysis categorizes genes from the most general to the most specific (same color indicates the same category), with the total number of genes in the pathway indicated, darker colors representing smaller *p* values, and the number of genes up-regulated or down-regulated in the pathway. The enrichment factor (the ratio of up-regulated or down-regulated genes to the total number of genes in the pathway) and the filtering *p* value and enrichment factor are also considered. Among the up-regulated DEGs, there is a significant enrichment in tissue structure and protein structure pathways, such as cardiac septum morphogenesis (GO:0060411), outflow tract morphogenesis (GO:0003151), cardiac septum development (GO:0003279), and negative regulation of protein-containing complex assembly (GO:0031333). In contrast, down-regulated DEGs predominantly enrich in pathways related to cytoarchitecture, such as the axoneme (GO:0005930) (Figure 1F).

### 3.3 DEGs interaction and gene expression analysis

**Figure 2:**
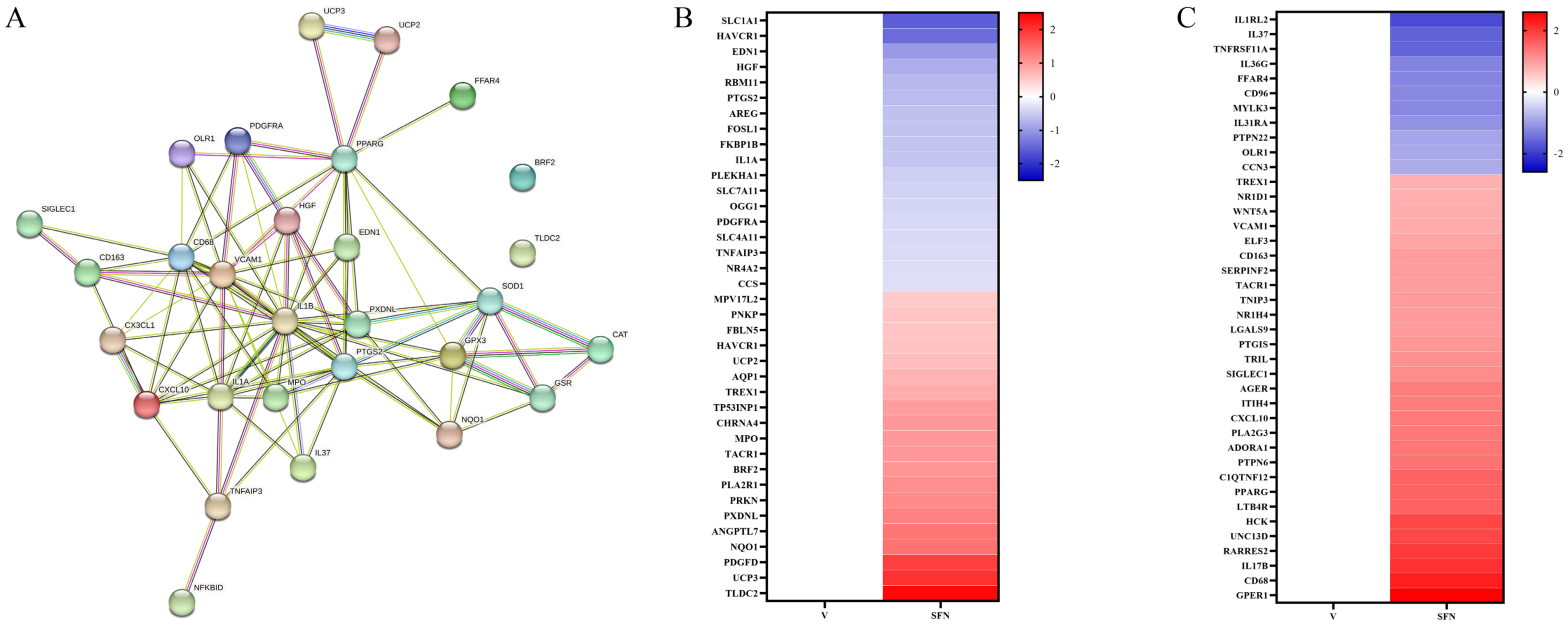
STRING analysis for gene correlation analysis and heat map associated with PPARγ. **(A)** Interactions between key genes involved in PPARγ transcriptional regulation of inflammatory response, oxidative stress, and NF-κB signaling pathway were predicted by STRING-ELIXIR analysis. Enriched *p* value: <1.0e-16. **(B)** Heat map of differentially expressed genes of the oxidative stress pathway. **(C)** Heatmap of differentially expressed genes of the inflammatory pathway.

In the present study, most of the differential genes in SFN-treated canine intestinal epithelial cells were centrally enriched in pathways related to oxidative stress response and inflammatory response compared with controls. In addition, PPARγ plays a role in inflammation and oxidative stress processes (17). Therefore, we selected key differential genes of oxidative stress response pathway, inflammatory response pathway, and NF-κB signaling pathway, and further STRING analysis predicted the relationship between these genes and PPARγ, and the results showed that they were highly correlated and interconnected by 93 edges. UCP2, UCP3, NQO1, and SOD1 interacted with PPARγ (Figure 2A). These data suggest that SFN may alleviate the inflammatory response of canine intestinal epithelial cells through PPARγ.

It has been demonstrated that the PPARγ gene is highly expressed in the intestine and exhibits a strong correlation with oxidative stress pathways (18). We conducted a screen of the expression values for differentially expressed genes associated with the oxidative stress pathway, normalized the data, and generated a heatmap (Figure 2B). Notably, the expression of antioxidant genes, including TLDC2 (19), NQO1 (20), BRF2 (21), and UCP2 (22) were significantly up-regulated. Furthermore, PPARγ is known to suppress inflammation via signaling pathways such as NF-κB (16). We plotted a heatmap of differentially expressed genes within the inflammatory signaling pathway. This revealed the downregulation of pivotal genes, such as TNFAIP3, which is known to suppress the NF-κB signaling pathway (23). Additionally, we observed an upregulation of the PPARγ gene, as illustrated in Figure 2C.

## 4 Discussion

The present study provides a comprehensive transcriptomic analysis of the effects of sulforaphane (SFN) on canine intestinal epithelial cells, revealing significant modulation of genes and pathways associated with inflammation, oxidative stress, and immune response. Our findings underscore the potential therapeutic implications of SFN in canine intestinal health and contribute to the broader understanding of its mechanisms of action.

The significant up-regulation of the PPARγ gene in response to SFN treatment is particularly noteworthy. PPARγ is a nuclear receptor with established roles in regulating inflammation and oxidative stress (24, 25). Its activation has been shown to suppress the production of pro-inflammatory cytokines and enhance the expression of antioxidant enzymes, thereby mitigating cellular damage. Our results align with previous studies that have highlighted the anti-inflammatory and antioxidant effects of SFN, suggesting that PPARγ may be a key mediator of these effects in canine intestinal epithelial cells.

The enrichment of differentially expressed genes in pathways related to lipid metabolism is another significant finding. SFN’s impact on lipid biosynthesis and catabolism could have implications for managing lipid-related disorders in dogs, such as pancreatitis and atherosclerosis. Further research is needed to explore the potential of SFN as a dietary supplement for the prevention and treatment of these conditions.

Our study also observed the down-regulation of genes associated with the cytokine-cytokine receptor interaction and MAPK signaling pathways. These pathways are crucial in the propagation of inflammatory responses (26, 27). The modulation of these pathways by SFN suggests a potential mechanism by which SFN could reduce inflammation in the canine intestine, supporting its use as an anti-inflammatory agent.

It is important to consider the translational relevance of our findings. The canine model is often used as a surrogate for human gastrointestinal research due to similarities in gut physiology and disease pathology (28). Therefore, our results may have broader implications for understanding the role of SFN in human intestinal health and its potential as a therapeutic agent in conditions such as inflammatory bowel disease.

However, our study is not without limitations. The *in vitro* nature of our experiments means that the effects of SFN on the whole organism are yet to be determined. Future studies should include *in vivo* models to validate our findings and explore the long-term effects of SFN on canine intestinal health. Additionally, the specific concentrations of SFN used in this study may not reflect dietary intake levels, suggesting a need for dose–response studies to establish optimal therapeutic dosages.

In conclusion, our transcriptomic analysis provides valuable insights into the molecular mechanisms by which SFN may exert its protective effects on canine intestinal epithelial cells. The modulation of key genes and pathways involved in inflammation, oxidative stress, and lipid metabolism suggests SFN’s potential as a therapeutic agent for promoting intestinal health in dogs (29). Further research is warranted to explore these findings *in vivo* and to translate these insights into clinical applications.

## Data Availability

The datasets presented in this study can be found in online repositories. The names of the repository/repositories and accession number(s) can be found below: https://www.ncbi.nlm.nih.gov/, PRJNA1130140.

## Footnotes

^1^ https://deweylab.github.io/RSEM/

^2^ http://metascape.org/

^3^ https://david.ncifcrf.gov/

^4^ http://www.bioinformatics.com.cn

^5^ https://cn.string-db.org/
